# Editorial: NK cells in viral immunology and immunotherapy

**DOI:** 10.3389/fimmu.2023.1216158

**Published:** 2023-05-23

**Authors:** Sachin Mulik, Pranay Dogra, Nabila Jabrane-Ferrat

**Affiliations:** ^1^ Department of Pulmonary Immunology, Center for Biomedical Research, The University of Texas Health Science Center at Tyler, Tyler, TX, United States; ^2^ Oncology, Genentech, South San Francisco CA, United States; ^3^ Institute for Infectious and Inflammatory Diseases (Infinity), Centre National de la Recherche Scientifique (CNRS) UMR5051, Institut National de la Santé et De la Recherche Médicale (INSERM) UMR1291, University of Toulouse III, Toulouse, France

**Keywords:** NK cells, viral infection, immunotherapy, cancer, immune response

Natural Killer (NK) cells are professional patrollers that constitute the first line of defense against pathogenic challenges, especially viruses, and cancer. These professional killers have the ability to restrict pathogens’ dissemination and cancer development thanks to the expression of a large panel of activating and inhibitory receptors. NK cells also produce a large array of soluble mediators to shape the immune response. Recent development in the field revealed that NK cells are able to establish immunological like-memory, a hallmark of adaptive immunity, to ensure protection against recurring infection and pathogen reactivation. A large body of evidence demonstrated that NK cell activities can be redesigned and enhanced for potential therapeutic applications beyond cancer and infection.

This Research Topic gathers advances on NK cell response to viral infection in order to provide insights into how different viruses shape NK cell phenotype and function, through collected articles focused on HCMV, HIV, HPV, Influenza A virus, and SARS-COV-2.

Many chemokine receptors, such as CXCR3, CXCR4, CXCR6, and their corresponding ligands are central for NK cell trafficking and function. For instance, the expression of CXCR6 on NK progenitors and immature NK cells is necessary for their egress from the bone marrow into blood and peripheral tissues. Analysis of the replenishment of the hematopoietic compartment after HSCT by Aviles-Padilla et al. reveals that NKG2C and CXCR6 are not co-expressed on NK cells. The NKG2C–CXCR6–, which constitutes the major subset of the post-transplant NK cell compartment, the CXCR6^+^, and the NKG2C^+^ are distinct NK cell subsets with unique phenotypic and functional features.

NK cell diversity during viral infection is underscored by the substantial changes in NK cell phenotype and functional plasticity, and in the proliferation and differentiation potential. Malengier-Devlies et al. report substantial changes in NK cell phenotype and functions in COVID-19 patients with a skewing toward secretory profile and a loss of cytotoxic function in patients with severe SARS-CoV-2 infection. In addition to an exhausted NK cell profile, severe COVID-19 was linked to the emergence of an NK cell subset with a platelet gene signature as a result of aggregates formation of NK cells with activated platelets. The link between patients’ genetic background and susceptibility to SARS-CoV-2 infection is far from being fully understood. In this regard, Hu et al. highlight the multifaceted role of NK cells in susceptibility versus protection. The analysis of the genetic background in Covid-19 patients from the early phases of the pandemic in Wuhan revealed that the expression of the inhibitory KIR2DL2 and its HLA-C ligand may increase the susceptibility to develop COVID-19 disease whereas the activating KIR2DL3 seems to have a protective role.

Functional diversity of NK cells may also underlie resistance to anti-viral therapies. Resistance to IFN-α and ribavirin treatment, in chronic HIV and HCV infection, has been clearly associated with the expansion of functionally skewed NK cells lacking the expression of CD56 (CD56^neg^ NK cells). Nonetheless, the role of CD56^neg^ NK cells in disease progression is far from being understood. To further define the features and functions of CD56^neg^ NK cells in the context of HIV infection, Cao et al. compared the phenotype and functions of the CD56^neg^ NK cells from HIV-infected individuals who are either treatment-naïve or ART-therapy responders. The authors revealed an accumulation of CD56^neg^ NK cell population characterized by impaired cytokine production and cytotoxic function (low amounts of IFN-γ, TNF-α, GzmB, and perforin) concomitant with high expression of the immune checkpoint mediators including the ectoenzyme CD39, the immunoreceptor tyrosine-based inhibitory domains (TIGIT) and CD95 in treatment-naive HIV patients. This study highlighted profound functional alterations to the NK cell compartment in HIV patients with an accumulation of dysfunctional CD56^neg^ NK cells.

NK cells have an inherent ability to recognize and kill cancer cells. However, their cytotoxic function is usually limited within the tumor microenvironment. The role of NK cells in the Juvenile-onset of recurrent respiratory papillomatosis (JO-RRP), chronic papillomatosis caused by vertical transmission or early exposure to Human papillomavirus (HPV6/HPV11), is still elusive. Wu et al. describe the phenotype and function of NK cells in Peripheral blood NK cells of JO-RRP patients characterized by decreased expression of the NKP30 and NKp46 activating receptors, and downregulation of cytotoxic function. Global unbiased analysis by RNA sequencing revealed a dysregulated NK cell cytotoxicity signature within JO-RRP tumors probably due to increased production of TGF-β1 production by papillomatosis. Further blockade of TGF-β1 ex-vivo using specific antibodies increased the expression of the NKp30 and NKG2D activating receptors and restored NK cell cytotoxic function. To further understand the clinical relevance of NK cells and their receptors in cancer development and progression, Xue et al. conducted an unbiased meta-analysis of 26 referenced studies of hepatocellular carcinoma. Interestingly, this study also highlighted the importance and receptors’ polyfunctionality of NK cells infiltrating the tumor bed. While the contribution of NK cell activating receptors is still unclear, this meta-analysis highlighted the role of inhibitory NK cell receptors as a predictive marker of tumor recurrence and attributed an advantageous prognostic value to NK cells expressing the maturation factor CD57. However, the precise role of NK cells expressing CD57 in HCC patients warrants further investigation. Furthermore, monitoring the expression level of NK cell activating receptors ligands by tumor cells could also serve as a possible prognostic marker.

For the longest time, immune memory has been viewed as the hallmark of the adaptive immune system. However, since the discovery of long-lived memory-like NK cells in 2006, their optimization as cellular immunotherapy for cancer has taken a new leap. Many strategies have been used to boost memory/adaptive NK cell antitumor responses. For instance, memory-like NK cells that differentiate upon brief cytokine activation (IL-12, IL-15, and IL-18) exhibit antitumor responses and have been used to treat leukemia. The review by Terren et al. highlights recent findings on the generation, functionality, and clinical applicability of cytokine-induced memory-like/adaptive human NK cells and discusses common features in comparison to other recent concepts of memory NK cells. Gao et al. also reviews the emergence of human NK cell subsets with memory-like/adaptive immune features under pathological conditions such as viral infection, transplantation, and cancer. NK cell responses against CMV, and the NK cell subsets provide arguments in favor of harnessing memory-like/adaptive NK for the development of promising strategies to treat cancer patients.

At the translational level, adoptive transfer of immune cells can be considered as a potential therapeutic approach to treat severe illness including viral infection. Gunasekaran et al. describe an original concept of an off-the-shelf human placenta hematopoietic stem cells-derived NK cells (CYNK cells) that can be used against viral infection. The authors provide evidence that CYMK cells recognize Influenza A virus-infected cells *via* specific receptor-ligand interactions, leading to NK cell degranulation and cytokine production. Although off-the-shelf NK cell products hold promise as anti-viral and anti-cancer immunotherapies for patients with very limited treatment options, further investigations are necessary before we can envision any real therapeutic breakthrough.

Half a century after the discovery of NK cells by Rolf Keissling and his colleagues and despite the growing interest in both basic and clinical aspects, several key elements of their function and mode of action remain to be unveiled. By presenting literature reviews and original data, this Frontiers in Immunology Topic on NK cell biology highlights new knowledge and key aspects of NK cell biology in viral infection and cancer. In addition, it touches on translational research to develop novel therapeutic perspectives.

## Author contributions

NJ-F is the main contributor to the drafted manuscript with contributions from others to produce the final version. All authors have made substantial contributions to the work and approved it for publication.

